# Dynamic EEG network responses to acupuncture stimulation in patients with stroke: a neurophysiological study

**DOI:** 10.3389/fnins.2026.1946735

**Published:** 2026-09-29

**Authors:** Jianxin Tan, Juanjuan Zhao, Chen Zhang, Xulong Fan

**Affiliations:** Department of Rehabilitation, Jiaozuo Hospital of Traditional Chinese Medicine, Jiaozuo, China

**Keywords:** acupuncture, electroencephalography, functional connectivity, neural plasticity, stroke

## Abstract

**Objective:**

Acupuncture may modulate brain activity through effects on the central nervous system. This study aimed to investigate short-term changes in spontaneous EEG activity and functional connectivity during acupuncture in patients with stroke using multichannel electroencephalography (EEG).

**Methods:**

Multichannel EEG recordings were collected at three stages: before acupuncture, during acupuncture, and after acupuncture. Acupuncture was applied at Jianyu, Quchi, and Waiguan. EEG activity was evaluated using power spectral density (PSD) analysis. Functional connectivity was assessed using the weighted phase lag index (wPLI). Functional brain networks were constructed for different frequency bands. Graph theory analysis was used to examine clustering coefficient and global efficiency across different network sparsities.

**Results:**

Power spectral density analysis showed significant stage-related changes in delta, theta, and alpha relative power, but not in beta power. Delta relative power decreased during acupuncture and partially recovered after acupuncture, but remained comparable to the pre-acupuncture level. Theta relative power increased during acupuncture and then decreased after acupuncture, with the post-acupuncture level lower than the pre-acupuncture level. Alpha relative power increased during acupuncture and decreased after acupuncture, but remained higher than the pre-acupuncture level. Whole-brain average wPLI showed a decrease in the delta band and an increase in the alpha band during acupuncture. No significant stage-related changes were observed in the theta-band average wPLI after correction for multiple comparisons. Graph-theoretical measures, including clustering coefficient and global efficiency, did not show statistically significant stage-related changes at the selected sparsity.

**Conclusion:**

Acupuncture was associated with short-term, frequency-dependent changes in spontaneous EEG activity and functional connectivity in patients with stroke. The observed changes were mainly reflected in spectral power and whole-brain average wPLI, while changes in overall network topology were not statistically supported. These findings provide preliminary neurophysiological evidence for the brain response to acupuncture stimulation.

## 1 Introduction

Stroke remains one of the leading causes of disability worldwide, presenting significant challenges in neurorehabilitation. It not only severely impairs patients’ personal lives but also places a heavy burden on their families and the healthcare system. Acupuncture, as an external neuromodulatory approach, has increasingly gained attention due to its potential to facilitate neural plasticity and recovery after stroke (Ke et al., 2024; Cong et al., 2025). Evidence suggests that acupuncture stimulation can modulate neural excitability, promote neurogenesis, angiogenesis, and foster cortical reorganization, thereby improving functional recovery (Xie et al., 2016; Qian et al., 2025).

Electroencephalography (EEG) is a valuable tool for assessing neural responses and alterations in functional connectivity following acupuncture (Hauck et al., 2017). Previous EEG-based studies have reported that acupuncture can significantly modulate spectral power in various frequency bands, particularly delta (0.5–4 Hz) and alpha (8–15 Hz) oscillations, associated respectively with neural inhibition/excitation balance and cognitive processing (Streitberger et al., 2008; Shen et al., 2025). Ericson et al. (2025) highlighted that alpha oscillations could serve as potential biomarkers of cognitive ability, reflecting cortical reorganization and plasticity.

Notably, acupuncture-induced EEG responses exhibit acupoint specificity, with distinctive patterns arising from different stimulation sites. Fang et al. (2009) demonstrated that acupuncture stimulation at various acupoints engages distinct neural networks involving limbic-paralimbic-neocortical regions, implying functional specificity of acupoints (Xu et al., 2021; Wang et al., 2023; Tian et al., 2024). Previous work by Yeo et al. (2012) also reported unique neural activation patterns elicited by acupuncture at GB34, linking specific acupoints to targeted neurological conditions (Yeo et al., 2012). As the He-Sea point of the Large Intestine Meridian, the Quchi acupoint has been associated with modulation of sensorimotor-related neural activity and alleviates numbness and weakness in the upper limbs (Xu et al., 2018). Research has shown that electroacupuncture at the Quchi and Zusanli acupoints can significantly improve neurological dysfunction scores in rats following ischemia (*p* < 0.05) and reduce the volume of cerebral infarction (Huang et al., 2017). Acupuncture at the Quchi point has been proven to be effective for anesthesia and chronic pain (Zhang et al., 2014; Huang et al., 2017). Acupuncture at the Waiguan point can produce sensations of soreness, numbness, distension, and heaviness, primarily activating the left temporal lobe and superior temporal gyrus (Huang et al., 2012). In a randomized sham-controlled study of post-stroke hemiplegic shoulder pain accompanied by subluxation of the shoulder joint, Sui et al. (2021) combined the acupoint Jianyu (LI14) with Binao (LI14), Jianzhen (SI9) and Jianliao (TE14), and applied electroacupuncture stimulation to these points; patients in the electroacupuncture group showed significant improvement in shoulder pain severity.

Existing literature on healthy individuals shows acupuncture at Zusanli (ST36) significantly increases delta power during stimulation but subsequently decreases after stimulation, whereas alpha power continuously rises during and post-stimulation (Yu et al., 2018). However, stroke-induced neural alterations may result in different EEG responses to acupuncture. Comparative research indicates altered functional connectivity and network patterns in stroke patients compared to healthy controls following acupuncture (Chen et al., 2024). For instance, Cho et al. (2013) observed differential network modulation through functional MRI following acupuncture in stroke patients versus healthy subjects (Chen et al., 2024).

Despite growing evidence, studies directly comparing EEG spectral and connectivity changes between healthy and stroke populations following acupuncture stimulation are limited. Furthermore, the neural mechanisms underlying distinct EEG spectral and network responses in stroke patients remain incompletely understood. Therefore, this study aims to investigate EEG spectral and functional connectivity alterations induced by acupuncture stimulation in stroke patients at Jianyu (LI15), Quchi (LI11), and Waiguan (SJ5) in stroke patients. Specifically, we hypothesize that stroke-induced neural disruption will manifest distinct EEG responses compared to healthy individuals, reflected in altered spectral power patterns and network connectivity dynamics. Our findings aim to deepen the understanding of acupuncture’s neuromodulatory mechanisms in stroke rehabilitation and provide neurophysiological evidence for understanding the immediate brain response to acupuncture stimulation after stroke.

## 2 Materials and methods

### 2.1 Participants

This study was designed as an exploratory neurophysiological investigation of the immediate EEG responses to acupuncture in patients with stroke. No formal *a priori* sample size calculation was performed. The initial sample size was determined mainly by the feasibility of participant recruitment and EEG data acquisition during the study period.

Stroke was diagnosed according to the Chinese Society of Neurology, Chinese Stroke Society (2018, 2019). The diagnosis was based on clinical neurological deficits and CT or MRI findings, with nonvascular causes excluded.

The inclusion criteria were: (1) a diagnosis of ischemic or hemorrhagic stroke according to the above criteria; (2) first-ever stroke; (3) age ≥ 18 years; and (4) motor dysfunction of the upper and/or lower limbs, including reduced muscle strength (grade 0–5) and/or ataxia.

The exclusion criteria were: (1) unstable vital signs; (2) large lobar or cerebellar hemorrhage (>30 mL in the cerebral hemisphere or >15 mL in the cerebellum), or intraventricular extension associated with hydrocephalus; (3) severe cardiovascular, respiratory, hepatic, renal, or other systemic diseases that could interfere with the study procedure; (4) other neurological diseases; (5) upper- or lower-limb dysfunction caused by other conditions, such as fracture or deformity; (6) inability to cooperate with the examination because of psychiatric disorders, cognitive impairment, visual impairment, or severe sitting-balance impairment; and (7) other conditions that could interfere with EEG recording or acupuncture, such as alcohol dependence, skull defects, or scalp defects.

Participants were withdrawn or considered dropouts if they voluntarily requested withdrawal, developed adverse events that prevented continuation, failed to complete the experimental procedure, or had EEG data of insufficient quality for analysis.

A total of 32 patients were initially enrolled. Two patients developed anxiety and syncope during acupuncture and could not complete the experimental procedure. They were therefore excluded from the final analysis. Finally, 30 patients were included in the analysis. The participant flow is shown in Figure 1.

**FIGURE 1:**
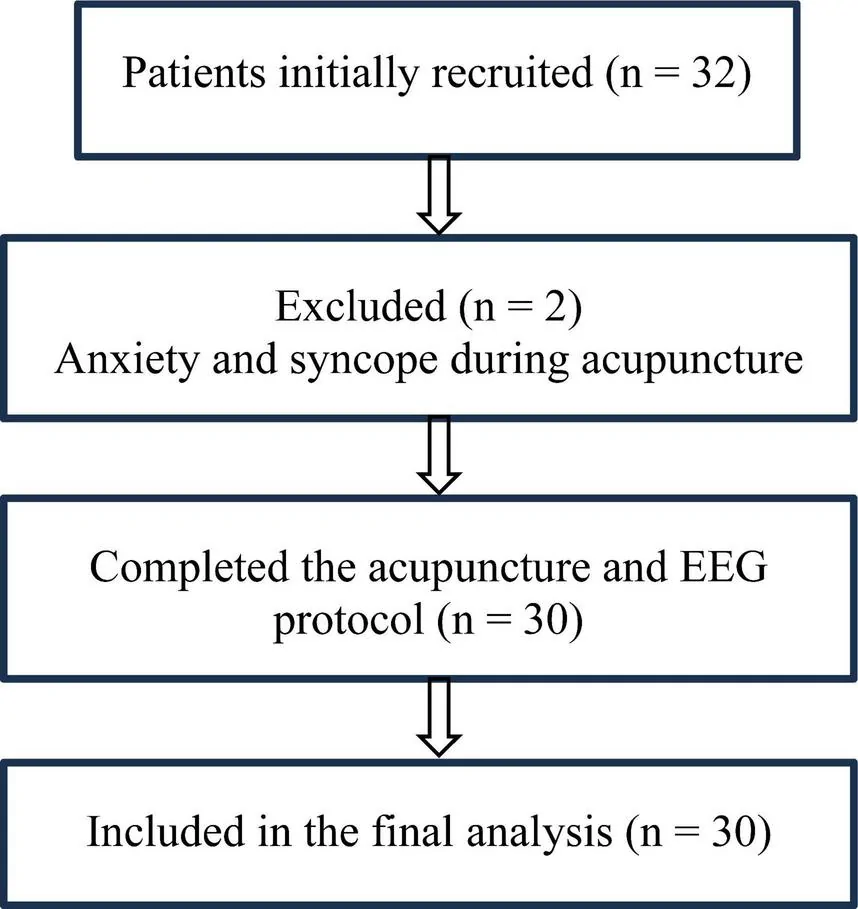
Flow diagram of participant recruitment and analysis.

All participants were right-handed and provided written informed consent before participation. The study protocol was approved by the Ethics Committee of Jiaozuo Hospital of Traditional Chinese Medicine (approval no. JZY-2024-01).

### 2.2 Acupuncture intervention

Acupuncture was performed at three acupoints on the upper limb: Jianyu (LI15), located at the depression anterior and inferior to the acromion; Quchi (LI11), situated at the lateral end of the transverse cubital crease; and Waiguan (SJ5), positioned two finger-widths proximal to the dorsal wrist crease between the radius and ulna. These points were selected based on their clinical relevance in stroke rehabilitation, as indicated by prior literature, highlighting their efficacy in modulating neurological functions and improving recovery outcomes. The three acupoints were stimulated sequentially rather than simultaneously. The order of stimulation was Jianyu, Quchi, and Waiguan. Manual lifting-thrusting manipulation was performed for 2 min at each acupoint, resulting in a total acupuncture manipulation period of approximately 6 min. After completion of the acupuncture procedure, the needles were removed. LI15, LI11, and SJ5 on the affected side were selected for acupuncture. These acupoints are commonly used in clinical practice for patients with stroke. The same acupoint combination was used for all participants to keep the stimulation relatively consistent.

The acupuncture procedure was standardized and conducted by an experienced and licensed acupuncturist. Participants were instructed to lie comfortably and remain awake with their eyes closed to minimize visual-related EEG activity. The protocol comprised three consecutive states: pre-acupuncture, acupuncture, and post-acupuncture. Initially, subjects maintained a resting state for 5 min (pre-acupuncture). Next, a sterile single-use Huatuo acupuncture needle (pure silver, 0.2 mm diameter, 40 mm length) was inserted into each acupoint at a depth of approximately 10 mm until participants reported the arrival of “De Qi”–a typical needling sensation indicating therapeutic efficacy. Jianyu (LI15), Quchi (LI11), and Waiguan (TE5) on the affected side were selected for acupuncture. The needles were inserted vertically at approximately 90°. The insertion depth was about 1–1.5 cun for Jianyu and Quchi and 0.5–1 cun for Waiguan. Following needle insertion, participants remained at rest for 2 min. Subsequently, a lifting-thrusting manipulation was performed for 2 min at each acupoint with a frequency of approximately 100 manipulations per minute (acupuncture). After manipulation, participants maintained a resting state for an additional 5 min (post-acupuncture). Finally, the needles were carefully removed, concluding the procedure.

### 2.3 EEG recording

Electroencephalogram (EEG) data were recorded continuously using a 32-channel EEG amplifier system (ZhenTec-NT1, Xi’an ZhenTec Intelligence Technology Co., Ltd., China). Ag-AgCl scalp electrodes were placed according to the international 10–10 electrode placement system. The EEG signals were digitized at a sampling rate of 500 Hz. To avoid artifacts associated with needle insertion and removal, a stable 3-min epoch from the middle of each state (pre-acupuncture, acupuncture, and post-acupuncture) was selected for subsequent analysis. EEG data preprocessing and analysis were conducted using MATLAB 2021b software.

### 2.4 EEG preprocessing

Continuous EEG was first filtered using a 50-Hz IIR notch filter with a Q factor of 1 and then a fourth-order Butterworth IIR band-pass filter from 2 to 45 Hz. Both filters were applied in the forward direction only. To reduce the influence of the initial filtering transient, the first 1,000 samples (2 s) were discarded after filtering.

The filtered data were divided into non-overlapping 500-sample (1 s) windows. Artifact rejection was performed before common-average referencing. If the absolute amplitude in any of the 30 scalp EEG channels exceeded 75 μV within a window, the complete 30-channel window was rejected. All retained windows were concatenated in chronological order and then re-referenced to the common average of the 30 scalp channels. No principal component analysis, independent component analysis, bad-channel identification, channel interpolation, or additional manual rejection was applied. Because the number of rejected windows differed among participants and stages, the final effective data length also varied.

### 2.5 EEG power spectral analysis

In this study, PSD analysis was performed to examine the distribution of EEG power across different frequency bands. The PSD was calculated using Welch’s method (Welch, 1967). EEG data were segmented using a Hamming window of 250 samples (500 ms), with an overlap of 125 samples (250 ms). FFT was applied to each segment, and the resulting periodograms were averaged to obtain the PSD. Relative PSD was calculated by dividing the power within each selected frequency band by the total EEG power within the 2–45 Hz frequency range, according to the equation below.

The FFT was then applied to each segment, and the resulting periodograms were averaged to obtain the PSD. The relative PSD of each frequency band was calculated by dividing the power of the specific band by the total power across all frequencies. Relative power was calculated for each frequency band by dividing the power within that band by the total EEG power between 2 and 45 Hz:


$$P\,o\,w\,e\,r_{r\,e\,l\,a\,t\,i\,v\,e}=\frac{\sum_{f=f\,1}^{f=f\,2}P\,o\,w\,e\,r\,\left(f\right)}{\sum_{f=f\,L}^{f=f\,H}P\,o\,w\,e\,r\,\left(f\right)}$$

In this equation, [*f*_1_, *f*_2_] represents the frequency range of the selected sub-band, whereas [*f*_*L*_,*f*_*H*_] represents the total frequency range used for normalization. In the present analysis, [*f*_*L*_,*f*_*H*_] = [2,45] Hz. The same normalization range was used for all participants and all three experimental stages.

### 2.6 Functional connectivity analysis

The weighted phase lag index (wPLI) was used to quantify consistent non-zero-phase-lag coupling between pairs of EEG channels and to reduce sensitivity to zero-lag effects associated with volume conduction (Vinck et al., 2011). For each participant, stage, and analyzed connectivity band (delta, theta, and alpha), the cross-power spectral density between channels x and y was estimated in MATLAB R2021b using the default settings of the cpsd function. For an effective input length of N samples, a Hamming window with a length of *L* = floor(N/4.5) samples was used, with floor(L/2) samples of overlap (approximately 50%). The number of discrete Fourier transform points was set to NFFT = max(256, 2*^n^*), where *n* = nextpow2(L). For the real-valued EEG signals sampled at 500 Hz, cpsd returned the one-sided cross-power spectrum. Because the sampling frequency was not explicitly supplied as an input argument to cpsd, its frequency vector was expressed as normalized angular frequency from 0 to π rad/sample; the corresponding physical frequencies were determined using the known sampling rate of 500 Hz. Because the effective data length could vary across participants and stages after artifact rejection, the resulting window length, number of overlapping samples, and potentially NFFT also varied accordingly rather than being fixed at a single set of values. Based on these values, the wPLI was calculated according to the following formula:


$$w\,P\,L\,I=\frac{\left|E\,\left[I\,m\,\left(C_{x\,y\,\left(f\right)}\right)\right]\right|}{E\,\left[\left|I\,m\,\left(C_{x\,y\,\left(f\right)}\right)\right|\right]}$$

where Cxy(f) denotes the cross-spectrum between channels x and y at frequency f, Im{ Cxy (f)} denotes its imaginary component, and the expectation is taken across spectral estimates within the frequency band of interest. The wPLI value ranges from 0 to 1, with larger values indicating more consistent non-zero-phase-lag coupling. This procedure generated a symmetric 30 × 30 channel-level wPLI matrix for each participant, stage, and analyzed band. Whole-brain average wPLI was calculated from the unique off-diagonal channel pairs and used as the participant-level connectivity outcome.

The wPLI ranges from 0 to 1, with higher values indicating more consistent non-zero phase-lag coupling between the two EEG signals. A value close to 0 indicates weak or inconsistent phase synchronization, whereas a value approaching 1 indicates highly consistent phase-lag coupling. Using this approach, a channel-by-channel synchronization matrix was generated, in which each element represented the wPLI value between two EEG channels.

The 30 × 30 wPLI matrix was used to describe functional connectivity across the selected frequency bands. To generate functional brain networks, proportional thresholding was applied over network densities ranging from 0.20 to 0.50. Since the average wPLI values differed among the three experimental conditions, an absolute threshold was not considered suitable for network construction. Therefore, proportional thresholding was adopted to maintain the same proportion of high-strength connections in each network and allow a more comparable assessment of network topology (Rubinov and Sporns, 2010; Zalesky et al., 2010; Quraan et al., 2013; van den Heuvel et al., 2017).

Network densities of 0.20–0.50 were examined in steps of 0.05. At each density, the corresponding proportion of the strongest connections was retained. For example, a density of 0.30 indicates that the strongest 30% of connections were retained. Network measures were examined across the full density range rather than selecting a single threshold based on statistical significance.

### 2.7 Graph theoretical analysis

In graph theory analysis, a brain network is generally represented as a collection of nodes and edges, where nodes refer to individual processing units and edges describe the connections or information interactions between them. The characteristic path length represents the average shortest distance between pairs of nodes, indicating the number of steps needed for information to travel across the network. This parameter is often used to assess the efficiency of global information communication.

Based on this concept, global efficiency can be calculated as the reciprocal of the characteristic path length. In addition, the clustering coefficient provides information about local connectivity patterns and reflects the degree of functional segregation within the network. Generally, networks showing higher global efficiency with lower clustering coefficients tend to display more random network characteristics, while networks with reduced global efficiency and increased clustering coefficients are more likely to demonstrate regular organization patterns.

### 2.8 Statistical analysis

Each binarized functional network comprised 30 electrode nodes and the retained undirected edges. The clustering coefficient quantified the tendency of neighboring nodes to form local triangles and was used as an index of local segregation. Global efficiency was calculated as the mean inverse shortest-path length across all node pairs and was used as an index of network-wide integration. Clustering coefficient and global efficiency were evaluated in the delta, theta, and alpha bands. Because normalized clustering and path length relative to matched random networks were not calculated, the present analysis did not test or establish small-world organization.

## 3 Results

### 3.1 Participant characteristics

Thirty patients with stroke were enrolled in this study. The demographic and clinical characteristics of the participants are presented in Table 1. The participants included 22 males and 8 females, with a mean age of 62.87 ± 5.17 years. The mean disease duration was 2.27 ± 0.39 months. Among the participants, 17 patients (56.7%) had ischemic stroke and 13 patients (43.3%) had hemorrhagic stroke. Regarding the affected side, 12 patients (40.0%) had left-sided lesions and 18 patients (60.0%) had right-sided lesions.

**TABLE 1:** Demographic and clinical characteristics of participants.

Characteristic	Value
Sample size	30
Sex (male/female)	22/8
Age (years)	62.87 ± 5.17
Disease duration (months)	2.27 ± 0.39
NIHSS	13.5 ± 2.40
Ischemic stroke	17 (56.7%)
Hemorrhagic stroke	13 (43.3%)
Left affected side	12 (40.0%)
Right affected side	18 (60.0%)

### 3.2 Relative power changes

As shown in Figure 2, stage had a significant effect on delta relative power, *F*(2, 58) = 89.770, *p*FDR = 7.00 × 10^–18^, η_p_^2^ = 0.756, and ηG^2^ = 0.680. Delta power decreased from Pre-Acup (0.4358 ± 0.0681) to Acup (0.1637 ± 0.0815), *t*(29) = 11.976, *p*Holm = 2.85 × 10^–12^, *dz* = 2.187, and then increased from Acup to Post-Acup (0.4028 ± 0.0810), *t*(29) = −10.681, *p*Holm = 2.89 × 10^–11^, *dz* = −1.950. Pre-Acup and Post-Acup did not differ after Holm correction (*p*Holm = 0.1240).

**FIGURE 2:**
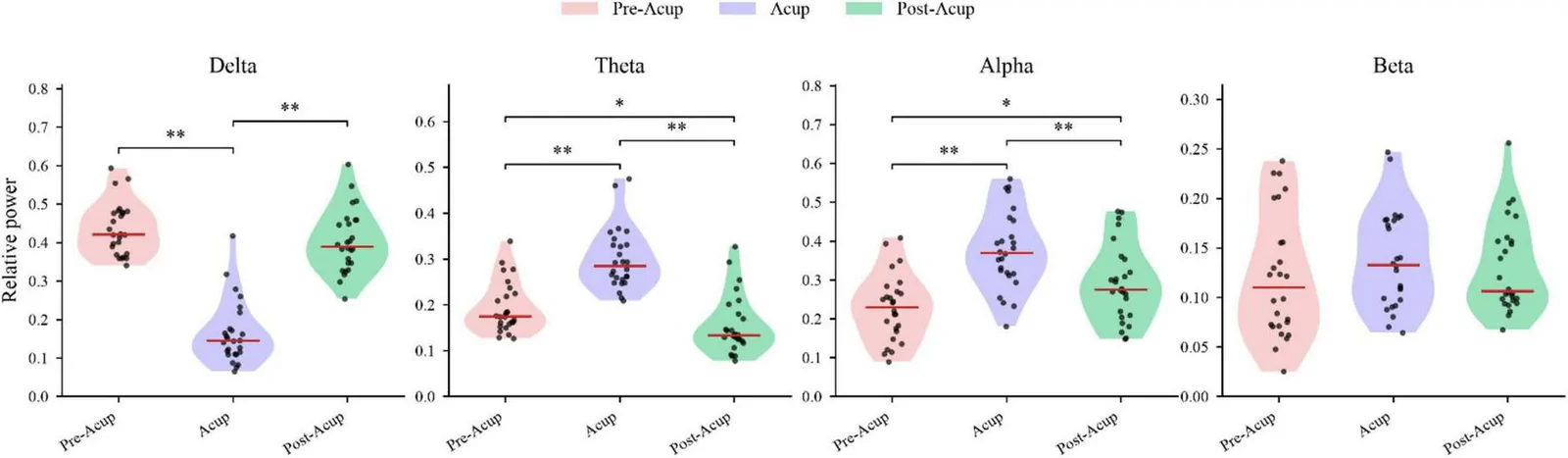
Dynamic changes in relative power across frequency bands during acupuncture stimulation. Relative power was calculated for delta (2–4 Hz), theta (4–8 Hz), alpha (8–15 Hz), and beta (15–30 Hz). Pink, purple, and green indicate Pre-Acup, Acup, and Post-Acup, respectively. Significance markers denote pairwise comparisons that passed the outcome-wise Holm correction after the corresponding frequency-band omnibus test passed domain-wise BH-FDR. Delta Pre-Acup versus Post-Acup was not significant and is not marked. *pHolm < 0.05; **pHolm < 0.01.

Theta relative power also showed a significant stage effect, *F*(2, 58) = 33.338, *p*FDR = 4.60 × 10^–10^, η_p_^2^ = 0.535, and ηG^2^ = 0.462. Theta power increased from Pre-Acup (0.1939 ± 0.0554) to Acup (0.2998 ± 0.0666), *t*(29) = −5.675, *p*Holm = 7.80 × 10^–6^, *dz* = −1.036, and decreased from Acup to Post-Acup (0.1554 ± 0.0634), *t*(29) = 7.275, *p*Holm = 1.55 × 10^–7^, *dz* = 1.328. Post-Acup theta power was also lower than Pre-Acup, *t*(29) = 2.371, *p*Holm = 0.0246, *dz* = 0.433.

Alpha relative power showed a significant stage effect, *F*(2, 58) = 20.523, *p*FDR = 2.43 × 10^–7^, η_p_^2^ = 0.414, and ηG^2^ = 0.278. Alpha power increased from Pre-Acup (0.2277 ± 0.0854) to Acup (0.3767 ± 0.1006), *t*(29) = −6.473, pHolm = 1.32 × 10^–6^, *dz* = −1.182, and decreased from Acup to Post-Acup (0.2912 ± 0.0991), *t*(29) = 3.578, *p*Holm = 0.00248, *dz* = 0.653. Nevertheless, Post-Acup alpha power remained higher than Pre-Acup, *t*(29) = −2.750, *p*Holm = 0.01016, *dz* = −0.502.

Beta relative power did not show a significant stage effect, *F*(2, 58) = 0.737, *p*FDR = 0.4829, η_p_^2^ = 0.025, and ηG^2^ = 0.016. Accordingly, no confirmatory *post hoc* comparison was performed. Overall, 8 relative-power contrasts met both the domain-wise omnibus BH-FDR criterion and the outcome-wise Holm criterion (Figure 2). Beta-band relative power therefore showed no statistically supported stage-related modulation under the present protocol. Because the study did not include a sham-acupuncture or time-matched resting control condition, this null beta-band result does not establish that the significant changes in other frequency bands were caused specifically by acupuncture rather than by nonspecific temporal or experimental factors.

### 3.3 Alterations in functional connectivity

The wPLI (weighted phase-lagged index) quantified consistent non-zero-phase-lag coupling between pairs of EEG channels on a scale from 0 to 1. Higher values indicate stronger consistent phase-lagged coupling but do not by themselves establish more efficient information transmission. For visualization, the matrix axes were grouped into five regions: prefrontal (PFC), frontal/central (FC), parietal/central (PC), temporal (TC), and occipital (OC). The heatmaps display connection strength only and do not represent channel-pair or region-wise significance.

As shown descriptively in Figure 3A, the delta, theta, and alpha wPLI matrices differed in their spatial distributions across the three stages. Delta connectivity appeared generally lower during acupuncture than before acupuncture, whereas alpha connectivity appeared generally higher during acupuncture. Theta matrices showed smaller and spatially heterogeneous changes. Because no inferential tests were performed for individual connections, regions, or heatmap cells, these spatial patterns were treated as descriptive and were not interpreted as evidence of region-specific significance or persistence.

**FIGURE 3:**
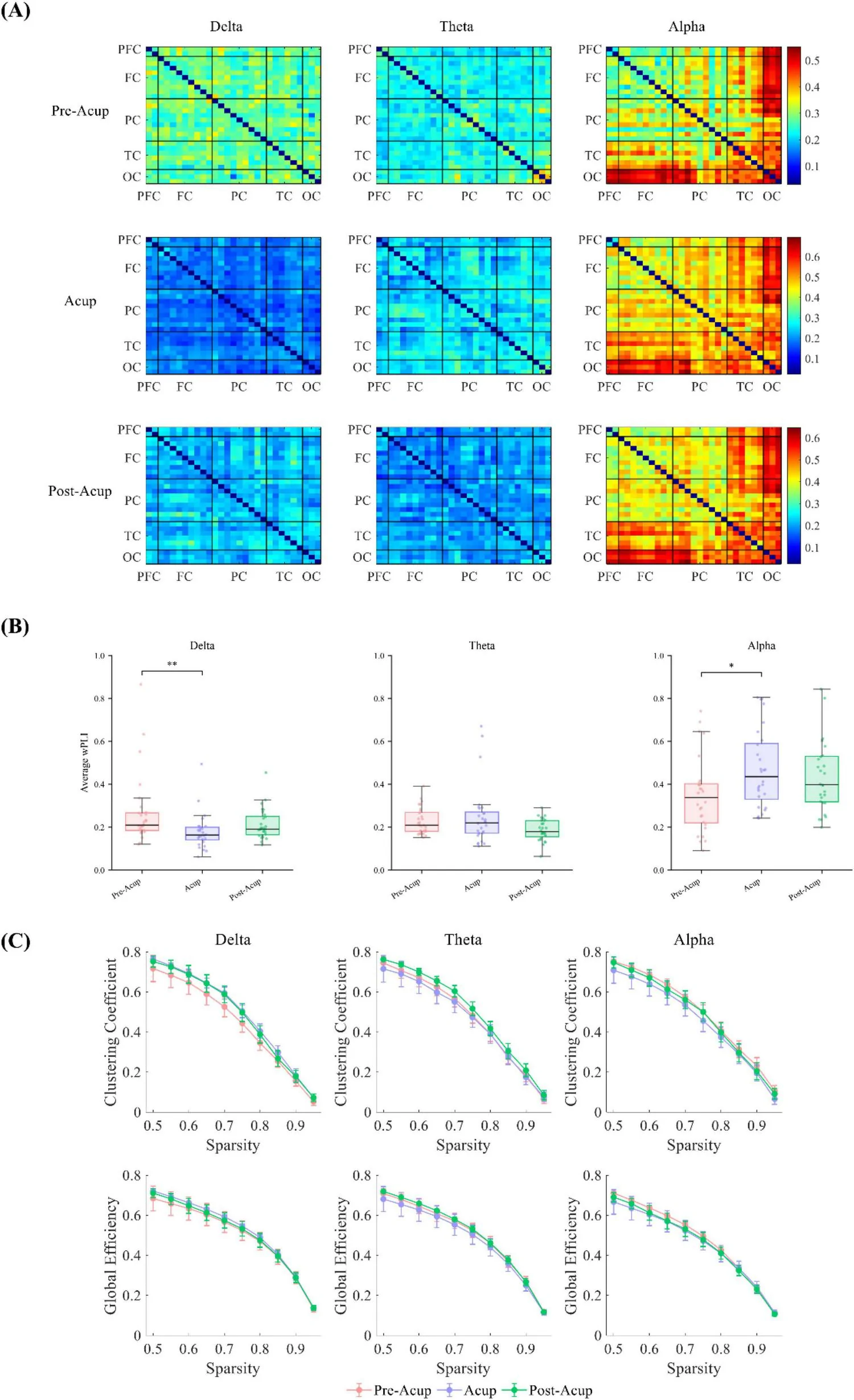
Effects of acupuncture on EEG functional connectivity and graph topological metrics in delta, theta, and alpha bands. **(A)** Group-level wPLI matrices for Pre-Acup, Acup, and Post-Acup. The color scale ranges from 0 to 1, with warmer colors indicating larger wPLI values. Matrix axes are grouped as follows: PFC, Fp1 and Fp2; FC, Fz, F3, F4, F7, F8, FCz, FC3, FC4, FT7, and FT8; PC, Cz, C3, C4, CPz, CP3, CP4, Pz, P3, and P4; TC, T3, T4, TP7, TP8, T5, and T6; and OC, Oz, O1, and O2. Heatmap cells are descriptive and do not indicate connection-wise significance. **(B)** Whole-brain average wPLI for the three stages (*n* = 30); where error bars are shown, they indicate SD. Significance markers are shown only for Delta Pre-Acup–Acup and Alpha Pre-Acup–Acup, which passed both domain-wise omnibus BH-FDR and outcome-wise Holm correction. **(C)** Clustering coefficient and global efficiency across network density 0.20–0.50, corresponding to sparsity 0.80–0.50. Density curves are descriptive; no separate inference was performed for individual density values. *Indicates *p* < 0.05, **indicates *p* < 0.01.

Whole-brain average wPLI showed frequency-specific stage effects (Figure 3B). The delta omnibus effect remained significant after BH-FDR correction within the three-outcome wPLI domain, *F*(1.429, 41.454) = 5.194, *p*FDR = 0.0471, η_p_^2^ = 0.152, and ηG^2^ = 0.096. Delta average wPLI decreased from Pre-Acup (0.2687 ± 0.1638) to Acup (0.1782 ± 0.0821), mean difference 0.0905 (95% CI, 0.0397–0.1412), *t*(29) = 3.646, *p*Holm = 0.00311, and *dz* = 0.666. No other delta pairwise contrast passed the outcome-wise Holm correction. Specifically, the Acup versus Post-Acup and Pre-Acup versus Post-Acup comparisons were not significant after Holm correction (*p*Holm = 0.0898 for both comparisons).

The alpha omnibus effect also passed domain-wise BH-FDR, *F*(2, 58) = 3.677, *p*FDR = 0.0471, η_p_^2^ = 0.113, and ηG^2^ = 0.078. Alpha average wPLI increased from Pre-Acup (0.3483 ± 0.1736) to Acup (0.4685 ± 0.1818), mean difference −0.1202 (95% CI, −0.2114 to −0.0290), *t*(29) = −2.696, *p*Holm = 0.0346, and *dz* = −0.492. No other alpha pairwise contrast passed the Holm correction. Specifically, the Acup versus Post-Acup (*p*Holm = 0.4930) and Pre-Acup versus Post-Acup (*p*Holm = 0.0717) comparisons were not significant after Holm correction. The theta omnibus effect did not pass domain-wise BH-FDR, *F*(1.314, 38.103) = 3.325, *p*FDR = 0.0653, η_p_^2^ = 0.103, and ηG^2^ = 0.070, and therefore no theta contrast was interpreted as confirmatory. The Holm-adjusted theta comparisons were Pre-Acup versus Acup, *p*Holm = 0.4811; Acup versus Post-Acup, *p*Holm = 0.1076; and Pre-Acup versus Post-Acup, *p*Holm = 0.0504. None of these theta comparisons was statistically significant after Holm correction.

Across network densities from 0.20 to 0.50 (sparsities 0.80–0.50), clustering coefficient and global efficiency showed small, frequency-dependent descriptive variation (Figure 3C). These curves were used to visualize threshold robustness only. No inferential test was performed at each density, and the curves did not support a conclusion of uniform improvement across frequencies or stages.

Taken together, the density-dependent curves showed small, frequency-dependent variations rather than a consistent increase in clustering coefficient or global efficiency across stages. These curves were therefore treated as descriptive visualizations of threshold dependence.

### 3.4 Changes in EEG functional network organization

Visualization of the wPLI-based EEG functional networks at sparsity 0.80 (network density 0.20) is presented in the connectivity-network panels of Figure 4. The circles represent the 30 EEG electrodes, and line color and thickness encode wPLI connection strength. The displayed networks showed frequency- and stage-dependent descriptive patterns; the edges were not tested individually and therefore do not indicate connection-level statistical significance.

**FIGURE 4:**
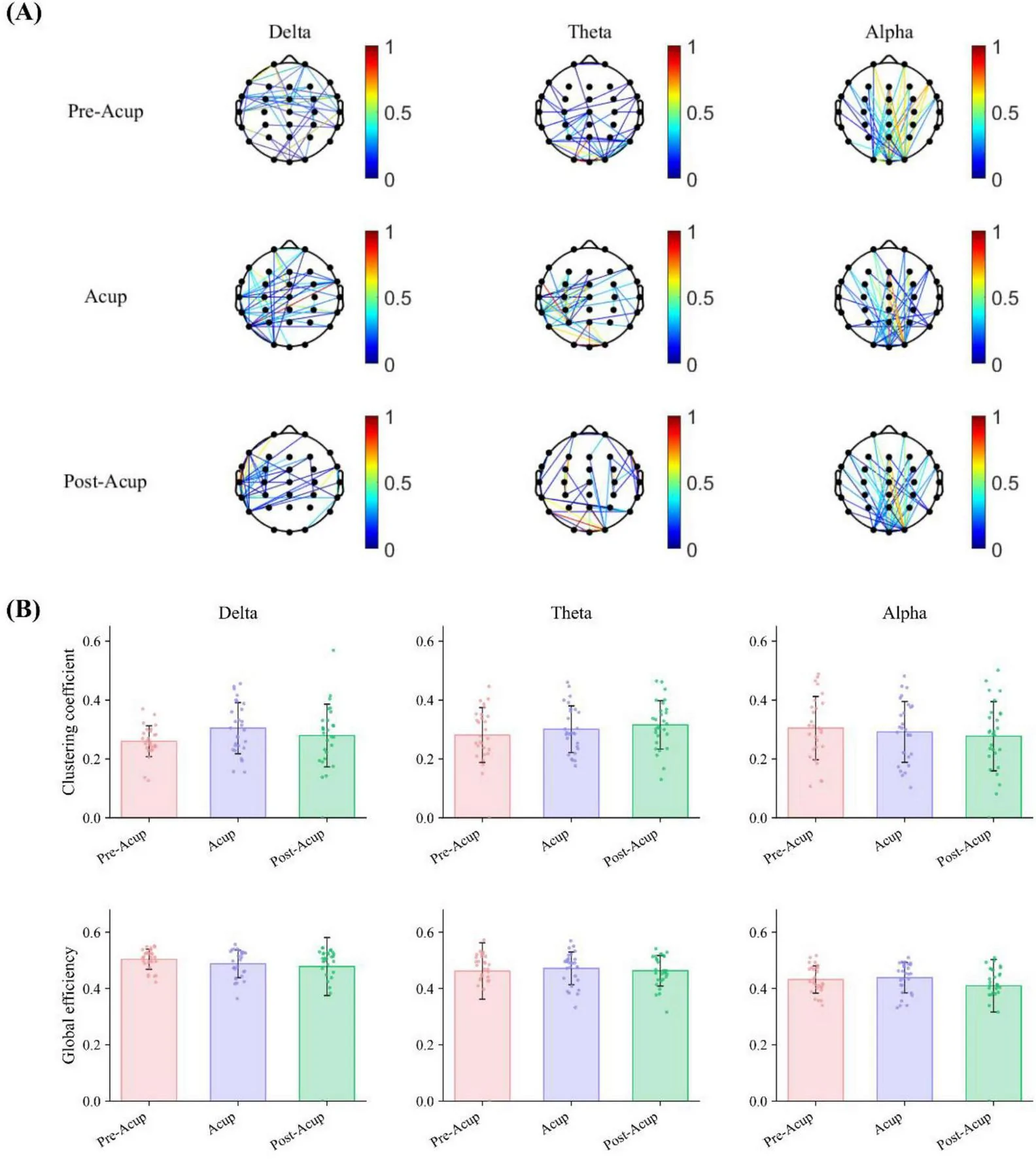
Graph-theoretical analysis of EEG functional networks across different frequency bands during acupuncture stages. **(A)** Functional networks at sparsity 0.80, corresponding to retention of the strongest 20% of possible undirected connections. Nodes represent the 30 scalp EEG electrodes. Edge color and thickness encode wPLI strength on a 0–1 scale and do not represent statistical significance. **(B)** Clustering coefficient and global efficiency at sparsity 0.80 for Pre-Acup, Acup, and Post-Acup (*n* = 30). Bars show group means, colored points show individual participants, and error bars show SD. None of the six graph-metric omnibus tests passed BH-FDR correction within the graph-metric domain; no significance bars or asterisks are shown, and the panels are interpreted descriptively.

At sparsity 0.80, none of the six graph-metric omnibus tests passed BH-FDR correction within the graph-metric domain (graph-metric panels of Figure 4). For clustering coefficient, the descriptive means ± SD for Pre-Acup, Acup, and Post-Acup were 0.2602 ± 0.0524, 0.3045 ± 0.0868, and 0.2796 ± 0.1066 in the delta band; 0.2812 ± 0.0932, 0.3009 ± 0.0793, and 0.3157 ± 0.0821 in the theta band; and 0.3047 ± 0.1073, 0.2920 ± 0.1033, and 0.2773 ± 0.1176 in the alpha band, respectively. The corresponding omnibus results were delta, *F*(1.649, 47.830) = 2.003, *p*FDR = 0.5267, η_p_^2^ = 0.0646, and ηG^2^ = 0.0432; theta, *F*(2, 58) = 1.066, *p*FDR = 0.5267, η_p_^2^ = 0.0354, and ηG^2^ = 0.0265; and alpha, *F*(2, 58) = 0.410, *p*FDR = 0.7985, η_p_^2^ = 0.0139, and ηG^2^ = 0.0101.

For global efficiency, the descriptive means ± SD for Pre-Acup, Acup, and Post-Acup were 0.5041 ± 0.0360, 0.4879 ± 0.0490, and 0.4782 ± 0.1030 in the delta band; 0.4623 ± 0.1002, 0.4717 ± 0.0581, and 0.4632 ± 0.0541 in the theta band; and 0.4321 ± 0.0485, 0.4388 ± 0.0539, and 0.4099 ± 0.0933 in the alpha band, respectively. The corresponding omnibus results were delta, *F*(1.435, 41.621) = 1.189, *p*FDR = 0.5267, η_p_^2^ = 0.0394, and ηG^2^ = 0.0232; theta, *F*(2, 58) = 0.148, pFDR = 0.8631, η_p_^2^ = 0.0051, and ηG^2^ = 0.0032; and alpha, *F*(1.613, 46.777) = 1.658, pFDR = 0.5267, η_p_^2^ = 0.0541, and ηG^2^ = 0.0316. Friedman sensitivity analyses led to the same conclusion (all graph-domain BH-FDR-adjusted *p* ≥ 0.6249; Kendall’s W ≤ 0.0754). Accordingly, all graph-metric differences were interpreted descriptively, and no pairwise significance bars or asterisks were shown in Figure 4.

## 4 Discussion

Previous studies have shown that post-stroke EEG spectral characteristics are typically marked by suppressed α/β rhythmic activity in the affected hemisphere, accompanied by enhanced δ rhythmic activity in the unaffected hemisphere (Iyer, 2017; Snyder et al., 2021; Bistriceanu et al., 2023). This interhemispheric imbalance is considered an important feature of altered brain activity after stroke and may be related to impaired functional connectivity and poor functional outcomes.

In the present study, multichannel EEG data were analyzed to examine changes in spontaneous brain activity during acupuncture stimulation. We focused on three aspects: spectral power, functional synchronization, and functional network organization. The results showed clear frequency-specific changes in EEG activity during acupuncture. Delta relative power decreased markedly during acupuncture and then increased after stimulation, approaching the pre-acupuncture level. Theta relative power increased during acupuncture but decreased after stimulation, and the post-acupuncture level was even lower than the pre-acupuncture level. In contrast, alpha relative power increased during acupuncture and decreased after stimulation, but remained higher than the pre-acupuncture level. No significant stage-related change was found in beta relative power. These findings suggest that acupuncture stimulation was associated with short-term changes in spontaneous EEG activity, but the direction and persistence of these changes differed across frequency bands. The changes in alpha activity may be of particular interest. Alpha oscillations are closely associated with motor and cognitive functions; their characteristic pattern-reduced power during task performance and increased power at rest-is interpreted as active inhibition of non-task-related brain regions, which helps optimize the allocation of neural resources (Pfurtscheller, 1992). Stroke patients often exhibit selective impairments in functional connectivity in the alpha band, particularly weakened local connections between key nodes in neural networks (Dubovik et al., 2012). In the present study, alpha relative power increased during acupuncture and remained above the pre-acupuncture level after stimulation. At the same time, whole-brain average alpha-band wPLI also increased during acupuncture. This combination may indicate that acupuncture was associated with enhanced alpha activity and phase synchronization during stimulation. However, the present study did not directly examine the relationship between these EEG changes and motor or cognitive recovery. Therefore, the observed alpha changes should be considered as neurophysiological responses rather than direct evidence of functional improvement.

Previous studies have suggested that interventions including transcranial direct current stimulation, mirror visual feedback, and transcranial alternating current stimulation may contribute to neurological recovery by regulating brain activity patterns. These approaches can influence interhemispheric balance through targeted enhancement of alpha activity or modulation of low-frequency mu rhythms (Liu et al., 2023; Tingting et al., 2025; Zhuang et al., 2025). The present findings show a partly similar pattern, particularly for alpha activity and synchronization. However, the current study should not be interpreted as demonstrating that acupuncture produces the same therapeutic effects as these neuromodulation approaches. Rather, the findings provide preliminary evidence that manual acupuncture may influence spontaneous EEG activity and functional synchronization in a frequency-dependent manner.

The wPLI results further showed different responses across frequency bands. Delta-band average wPLI decreased during acupuncture and partially recovered after stimulation, whereas alpha-band average wPLI increased during acupuncture. The theta-band omnibus effect did not remain significant after correction. These findings suggest that the effect of acupuncture on functional synchronization was not uniform across frequency bands. The decrease in delta synchronization may reflect a temporary change in low-frequency functional interactions during stimulation, while the increase in alpha synchronization may indicate a different neural response. However, these interpretations remain tentative because wPLI measures functional synchronization rather than direct anatomical or causal connections.

Functional brain networks were also reconstructed to further examine network organization. The connectivity matrices showed visible differences in spatial distribution across the three stages, especially in the delta and alpha bands. However, these patterns were descriptive because no statistical tests were performed for individual connections or network regions. More importantly, none of the six graph-metric omnibus tests showed a significant stage effect after correction. Therefore, the changes in clustering coefficient and global efficiency should be interpreted as descriptive variations rather than evidence of improved network segregation or global information integration. The absence of significant graph-theoretical changes also suggests that changes in spectral power and average functional synchronization do not necessarily translate into measurable changes in global network topology under the present experimental conditions.

The frequency-specific findings may partly explain why different EEG measures showed different responses to acupuncture. Delta and theta activity showed relatively transient changes, with both measures returning toward or below their pre-acupuncture levels after stimulation. Alpha activity showed a more persistent response, with both relative power and average wPLI remaining elevated after acupuncture compared with the pre-acupuncture condition. This pattern suggests that different oscillatory systems may respond to acupuncture on different time scales. However, the present study only examined short-term EEG changes around a single acupuncture session. Therefore, it remains unclear whether these changes are transient physiological responses or whether repeated acupuncture may lead to longer-term changes in neural plasticity.

Manual acupuncture was performed at the “Jianyu,” “Quchi,” and “Waiguan” acupoints, and changes in EEG power across different frequency bands were observed. The results suggested that acupuncture could influence the distribution of EEG power, although the effects may vary depending on the stimulated acupoint. Previous studies have reported that acupuncture at the “Quchi” acupoint increased EEG power in low-frequency ranges (0.5–12 Hz), whereas stimulation at the “Sishencong” acupoint was associated with increased power in the high-frequency range (12–30 Hz) and reduced power in low-frequency bands.

It has been demonstrated that EEG power change was associated with nerve activity, which could be inhibited or excited by acupuncture (Cong et al., 2025). Additionally, some studies showed that acupuncture on non-acupoints had much stronger effects on the power of EEG than acupoints, as stimulation on these points might cause tension, stress, and pain in the surrounding muscles, which could affect the EEG signal (You et al., 2011, 2013). Here, we selected the “Jianyu,” “Quchi,” and “Waiguan” acupoints because they are relatively easy to locate and are commonly used in clinical practice. The same acupoint combination was used for all participants to keep the stimulation protocol consistent. The present findings should therefore be considered specific to this acupuncture protocol, rather than generalized to acupuncture at other acupoints.

Several limitations should also be considered. First, the study did not include a sham-acupuncture or time-matched resting control condition. Therefore, the observed EEG changes cannot be attributed specifically to acupuncture, as nonspecific factors such as time, sensory stimulation, or experimental procedures may also have contributed. Second, the study focused mainly on short-term EEG responses and did not collect post-treatment clinical scale scores. Thus, the relationship between the observed EEG changes and clinical functional recovery could not be evaluated. Third, although changes in functional networks were observed descriptively, the graph-theoretical metrics did not show significant stage-related differences after correction. Future studies should combine EEG recordings with clinical assessments and include appropriate control conditions. Longitudinal designs may also help determine whether the short-term EEG changes observed here are related to longer-term neural plasticity and functional recovery after repeated acupuncture.

## 5 Conclusion

In patients with stroke in the non-acute phase, acupuncture was associated with short-term changes in spontaneous EEG activity and functional connectivity. Delta, theta, and alpha relative power showed significant stage-related changes, while beta power did not. Delta-band average wPLI decreased during acupuncture, whereas alpha-band average wPLI increased. These changes were partly reversed or maintained after acupuncture, depending on the frequency band. In contrast, graph-theoretical measures, including clustering coefficient and global efficiency, did not show statistically significant stage-related changes at the selected sparsity. These findings suggest that the immediate EEG response to acupuncture is frequency-dependent, but the present data do not provide sufficient evidence for a significant change in overall network topology.

Several limitations should be considered. First, the sample size was relatively small, and no *a priori* sample size calculation was performed. The findings should therefore be regarded as exploratory. Second, there was no sham-acupuncture or time-matched resting control condition. Thus, the observed EEG changes cannot be attributed specifically to acupuncture, as nonspecific temporal or experimental effects cannot be excluded. Third, post-treatment clinical scale scores were not collected, so the relationship between the observed EEG changes and actual functional recovery remains unclear. In addition, only one acupuncture protocol was examined. Finally, the present analysis focused mainly on individual frequency bands, and interactions between frequency bands were not investigated. Larger controlled studies with longitudinal EEG and clinical assessments are needed to further examine the relationship between acupuncture, brain activity, and post-stroke recovery.

## Data Availability

The raw data supporting the conclusions of this article will be made available by the authors, without undue reservation.
